# Binding and Activating of Analgesic Crotalphine with Human TRPA1

**DOI:** 10.3390/membranes15060187

**Published:** 2025-06-19

**Authors:** Mingmin Kang, Yanming Zhang, Xiufang Ding, Jianfu Xu, Xiaoyun Pang

**Affiliations:** 1 State key Laboratory of NBC Protection for Civilian, Beijing102205, China; comingk0123@163.com (M.K.); messi10_zhang@163.com (Y.Z.); 13552909516@163.com (X.D.); 2 Key Laboratory of Biomacromolecules (Chinese Academy of Sciences), National Laboratory of Biomacromolecules, Institute of Biophysics, Chinese Academy of Sciences, Beijing 100101, China

**Keywords:** crotalphine, TRPA1, binding site, cryo-EM, structural comparisons

## Abstract

TRPA1 (Transient Receptor Potential Ankyrin 1), a cation channel predominantly expressed in sensory neurons, plays a critical role in detecting noxious stimuli and mediating pain signal transmission. As a key player in nociceptive signaling pathways, TRPA1 has emerged as a promising therapeutic target for the development of novel analgesics. Crotalphine (CRP), a 14-amino acid peptide, has been demonstrated to specifically activate TRPA1 and elicit potent analgesic effects. Previous cryo-EM (cryo-electron microscopy) studies have elucidated the structural mechanisms of TRPA1 activation by small-molecule agonists, such as iodoacetamide (IA), through covalent modification of N-terminal cysteine residues. However, the molecular interactions between TRPA1 and peptide ligands, including crotalphine, remain unclear. Here, we present the cryo-EM structure of ligand-free human TRPA1 consistent with the literature, as well as TRPA1 complexed with crotalphine, with resolutions of 3.1 Å and 3.8 Å, respectively. Through a combination of single-particle cryo-EM studies, patch-clamp electrophysiology, and microscale thermophoresis (MST), we have identified the cysteine residue at position 621 (Cys621) within the TRPA1 ion channel as the primary binding site for crotalphine. Upon binding to the reactive pocket containing C621, crotalphine induces rotational and translational movements of the transmembrane domain. This allosteric modulation coordinately dilates both the upper and lower gates, facilitating ion permeation.

## 1. Introduction

Transient receptor potential (TRP) channels are considered to be one of the largest superfamilies of ion channels, which are ubiquitously expressed and functionally diverse across eukaryotic organisms [1]. Canonical TRP channels are tetramers and are phylogenetically classified into seven subfamilies, comprising a total of 28 distinct channel proteins in mammals. Among these, transient receptor potential Ankyrin 1 (TRPA1) is the sole member of the TRPA subfamily present in mammals [2,3,4,5].

TRPA1 is a non-selective cation channel localized on the plasma membrane, which exhibits permeability to various cations, including sodium (Na^+^) and calcium (Ca^2+^) ions [6,7]. It is widely expressed in sensory neurons of the dorsal root, trigeminal, and nodose ganglia, where it functions as a nociceptor and plays a role in mediating pain and inflammation perception [8,9,10,11,12]. Emerging as a promising therapeutic target for pain management, substantial evidence indicates that both antagonizing the TRPA1 ion channel and desensitizing its activity represent effective strategies for alleviating TRPA1-mediated pain [13,14,15,16].

TRPA1 can be activated by a variety of irritant chemical substances, classifiable into two major categories: electrophilic compounds and non-reactive compounds [8,17,18]. Electrophilic compounds activate TRPA1 by covalently modifying specific cysteine residues in its cytoplasmic N-terminal domain. For instance, electrophilic compounds benzyl isothiocyanate (BITC) and JT010 primarily bind to C621, while iodoacetamide activates TRPA1 through interactions with both C621 and C665 in the N-terminal domain [19,20,21,22,23,24]. In contrast, non-reactive compounds induce activation through non-covalent intermolecular interactions, as exemplified by GNE551 that binds through polar contacts [25,26].

Crotalphine (CRP), a toxin containing 14 amino acid residues isolated from the venom of the South American rattlesnake Crotalus durissus terrificus, has been proven to have effective and persistent antinociceptive effect [27,28,29,30]. Unlike opioid drugs, repeated administration of crotalphine does not induce tolerance, which further emphasizes the therapeutic potential of this peptide in treating pain [28]. Crotalphine has been demonstrated to act as an agonist of TRPA1 channels, specifically activating and strongly desensitizing the TRPA1 ion channel. Importantly, the targeting of TRPA1 by crotalphine and the subsequent desensitization are closely linked to its analgesic effects [31]. The direct activation of TRPA1 by crotalphine and its subsequent desensitization highlight its potential role in modulating TRPA1-mediated signaling pathways. Investigating the binding mode of crotalphine to TRPA1 is of significant importance, as it not only holds the potential to unravel the intricate mechanisms underlying their interaction but also provides critical insights for the development of novel TRPA1-targeting therapies. However, the precise binding site of crotalphine on TRPA1 and the detailed molecular mechanisms governing their interaction remain unresolved. Therefore, this study aims to identify the specific binding site of crotalphine on TRPA1, offering key clues to reveal the complex mechanisms of their interaction. Our data clearly demonstrate that C621 is the binding site for crotalphine in the human TRPA1 ion channel.

## 2. Methods

### 2.1. Protein Expression and Purification

cDNA of human TRPA1 was cloned into the home-made SmartBM vector (Patent No. ZL 2023 1 1601025.2), with an EGFP-tag followed by twin-Strep Tag at C-terminal. Baculovirus was generated according to the manufacturer’s protocol (Bac-to-Bac, Invitrogen, CA, USA) and amplified in SF9 cells at 28 °C. Then, hTRPA1 was expressed in HEK293F cells under the conditions of 37 °C and 8% CO_2_. After 12–16 h, 10 mM sodium butyrate was added to the cell culture. After an additional 24 h, the cells were harvested by centrifugation at 1000× *g* for 10 min and frozen in liquid nitrogen for further protein purification.

Cells were thawed and resuspended in lysis buffer (50 mM Tris pH 8.0, 150 mM NaCl, 10% glycerol, 1 mM inositol hexaphosphate (InsP_6_), 1 mM TCEP, 100 mU/mL, protease inhibitor) at 4 °C. The cell suspension was centrifuged at 35,000 rpm for 45 min at 4 °C to collect the cell pellets. The cell pellets were resuspended in lysis buffer and the lysate was incubated with a final concentration of 1% (*w*/*v*) LMNG and 0.2% (*w*/*v*) CHS at 4 °C for 2 h. Subsequently, the supernatant was collected by centrifugation at 35,000 rpm for 45 min and 10% (*w*/*w*) biolock was added. The supernatant was then incubated with Strep-Tactin resin at 4 °C for 2 h. The mixture was transferred to a gravity column and washed with 30 column volumes of a washing buffer (50 mM Tris pH8.0, 150 mM NaCl, 1 mM InsP_6_, 1 mM TCEP, 0.005%LMNG, 0.001%CHS). TRPA1 protein was eluted with an elution buffer (50 mM Tris pH8.0, 150 mM NaCl, 100 mM biotin, 1 mM InsP_6_, 0.5 mM TCEP, 0.005%LMNG, 0.001%CHS).

The TRPA1 protein sample was concentrated by ultrafiltration and subsequently incubated overnight with a quantity of PMAL-C8 that was three times the amount of the protein. After adding bio-beads to the protein sample and mixing at 4 °C for 2 h, the beads were removed. Then TRPA1 protein was further purified by size exclusion chromatography (SEC) using Superose 6 increase 10/300 GL (Cytiva, Uppsala, Sweden) within a buffer solution (comprising 50 mM Tris at pH 8.0, 150 mM NaCl, 1 mM InsP_6_, and 0.5 mM TCEP). The fractions containing the target protein were collected and concentrated to an approximate concentration range of 3–6 mg/mL. The concentrated protein was then incubated with 100 μM crotalphine at 4 °C for 10 min before preparing the cryo-EM sample.

### 2.2. Cryo-EM Sample Preparation and Data Acquisition

A total of 3 μL of the incubated complex sample was applied to a glow-discharged Au grid (Quantifoil, Au R1.2/1.3, 300 mesh) to prepare a cryo-EM sample using Vitrobot Mark IV (Thermo Fisher, Waltham, MA, USA). The datasets were collected on a 300 kV Titan Krios (Thermo Fisher) transmission electron microscope equipped with a K2 camera (Gatan, Pleasanton, CA, USA). Utilizing the Serial EM 4.1 software, movies were recorded. Each movie contained 32 frames, with a pixel size precisely measured at 1.04 Å. The collection was carried out with an accumulated dose of 60 e/Å^2^. Defocus value was set within the range of −1.0 to −2.0 μm.

### 2.3. Cryo-EM Data Processing

For the processing of cryo-EM data, all the major steps were carried out using cryoSPARC 3.2 [32]. After performing CTF estimation on the collected micrographs, high-quality micrographs suitable for further image processing were obtained. We used the particle template generated from the previously published TRPA1 map (EMD-21129) to perform particle picking.

For TRPA1, a total of 6,123,453 particles were picked from micrographs and extracted (with 2 × 2 Fourier binning and a box size of 128 pixels). After one round of 2D classification, 21 classes that showed clear secondary structure features of TRPA1 were selected. The previously published TRPA1 map (EMD-21128, low-pass filtered to 20 Å) was used as the initial volume for Heterogeneous Refinement. After one round of Heterogeneous Refinement, 482,970 particles were re-extracted and un-binned (with a box size of 256 pixels) and then subjected to three additional rounds of Heterogeneous Refinement. One class, which contained 148,984 particles and displayed the most high-resolution features of the channel, was selected and subjected to Homogeneous Refinement. This process generated a map with a resolution of 3.08 Å, as estimated by the “gold-standard” Fourier shell correlation (FSC) at the 0.143 criterion. The resolution was further increased to 3.07 Å after Non-Uniform Refinement in CryoSPARC 3.2 (PDB: 9M8S).

For TRPA1-crotalphine complex, a total of 10,750,795 particles were extracted from micrographs after being picked, with 2 × 2 Fourier binning applied and a box size of 128 pixels, and then one round of 2D classification was performed. A total of 1,420,193 particles were selected for the next round of Heterogeneous Refinement, and the initial volume model used for the complex is the same as the one used for heterogeneous refinement of TRPA1. A total of 424,524 particles were extracted again, with a box size of 256 pixels, and then subjected to three more rounds of Heterogeneous Refinement. One class that contains 203,880 particles was selected for Homogeneous Refinement, and it generated a map with a resolution of 3.96 Å. The resolution was further improved to 3.83 Å through Non-Uniform Refinement. Local Refinement was improved to 3.81 Å (PDB: 9M8N).

### 2.4. Model Building

To construct the atomic models, we utilized two TRPA1 structures as references: the ligand-free TRPA1 structure with bound calcium (PDB 6V9W, solved in LMNG) for developing the apo TRPA1 model, and the iodoacetamide-modified TRPA1 structure (PDB 6V9X, solved in PMAL-C8) for creating the TRPA1-crotalphine complex model. The structure was refined manually in Coot [33] and was refined automatically using the Phenix-real_space_refine function [34]. Finally, the model was further refined by Phenix.

### 2.5. Patch-Clamp Electrophysiology in HEK293 Cells

HEK 293T cells were transiently transfected with human TRPA1 (hTRPA1)-encoding plasmids. Twenty four hours after transfection, the GFP-positive cells were patch-clamped at room temperature (25 °C) with a HEKA EPC10 single patch clamp amplifier (HEKA, Lambrecht, Germany). The currents of TRPA1 were recorded in the whole cell. After obtaining electrical access, a 200 ms voltage ramp from −120 mV to +120 mV followed by a return to the holding potential of −80 mV was applied throughout all compound addition periods to measure the current of TRPA1. For analysis, the currents measured at +120 mV were used. Cell membrane capacitance was recorded directly from the amplifier following whole-cell access. Current density was calculated as the current amplitude divided by the cell membrane capacitance. The voltage protocol was applied every 10 s during the experimental recording period. The extracellular solution contained 137 mM NaCl, 4 mM KCl, 10 mM Glucose, 10 mM HEPES, 1 mM MgCl_2_, 2 mM CaCl_2_, pH 7.4. The intracellular medium consisted of 140 mM CsCl, 1 mM MgCl_2_, 10 mM HEPES, 5 mM EGTA, 2.6 mM CsCl, pH 7.2. All chemicals were of analytical grade.

### 2.6. Microscale Thermophoresis (MST)

The equilibrium dissociation constant (Kd) value of crotalphine with respect to the wild-type and mutant of hTRPA1 was determined using the Monolith NT.115 instrument produced by NanoTemper (Munich, Germany). First, 10 μL of the target protein with a concentration of 50 nM and an equal volume of crotalphine with an initial concentration of 4 μM were incubated on ice for 5 min. Then, the incubated sample was loaded into capillaries and placed into the instrument for the assessment of the binding affinity. Due to the EGFP fluorescent tag on the protein sample, the nano-blue channel was directly chosen for measurement. The measurements were carried out at 22 °C with 10% LED power and medium MST power. The data were analyzed using Nano-Temper Analysis 2.3 software.

## 3. Results

### 3.1. The Function and Structure of TRPA1

**Figure 1:**
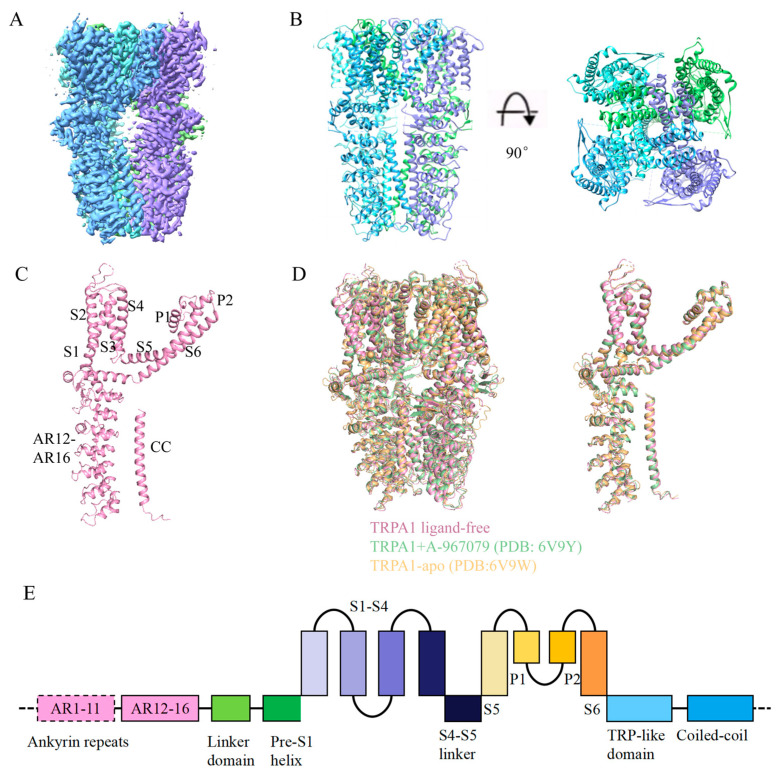
Structure of ligand-free TRPA1. (**A**) The cryo-EM reconstruction map of ligand-free TRPA1. The four subunits are shown in green, blue, cyan and purple. (**B**) The structural model of ligand-free TRPA1. The four subunits are shown in green, blue, cyan and purple. (**C**) The structure of a single TRPA1 subunit with six transmembrane α-helices (S1–S6), two pore helices (P1, P2), coiled coil (CC), and five ankyrin repeat domains (AR12–AR16). (**D**) Structural superposition of the ligand-free TRPA1 with previously reported TRPA1 structures (PDB: 6V9Y, 6V9W). The ligand-free TRPA1 is shown in pink, 6V9W in orange, and 6V9Y in green. (**E**) A linear diagram depicting the major structural domains of TRPA1.

Full-length human TRPA1 protein was expressed in HEK293F cells and purified to homogeneity (Figure S1B,C). Samples were prepared by flash-freezing and imaged through cryo-EM. Finally, we determined 3D structures of the ligand-free human TRPA1 at a resolution of 3.1 Å (Figure 1A and Figure S2A,B). The TRPA1 structure we obtained is consistent with previous cryo-EM studies [22,23,35]. TRPA1 is homotetramer; each subunit is composed of six transmembrane α-helices (S1–S6), two pore helices (P1, P2) between the helices S5 and S6, and the large intracellular N-terminal and C-terminal. Four identical subunits interact with each other to form the central ion permeation pore, below which the coiled coil (CC) is located. The N-terminal five ankyrin repeat domains (AR12–AR16) surround CC and connect with the AR1-AR11. Consistent with previous results, our structure is also insufficient to determine the structure of AR1-AR11 (Figure 1B,C,E). The ligand-free structure of TRPA1 is highly similar to the previously resolved ligand-free structure (PDB: 6V9W) [23], with a root mean square deviation (RMSD) of 1.021 Å for 506 Cα atoms. Furthermore, by comparing the ligand-free TRPA1 structure with the previously determined closed-state TRPA1 structure bound to the antagonist A-967079 (A-96) (PDB: 6V9Y) [23], we found that the overall structures of ligand-free and A-96-bound TRPA1 are highly similar, with an RMSD of 0.806 Å for 536 Cα atoms. Structural comparison of the pore dimensions revealed that both the upper and lower gates in the ligand-free TRPA1 structure maintain constricted apertures incompatible with ion permeation (upper: 8.1 Å; lower: 5.4 Å). These observations indicate that the conformation of our ligand-free TRPA1 adopts a closed-state conformation (Figure 1D).

### 3.2. Cryo-EM Structure of TRPA1-Crotalphine Complex

**Figure 2:**
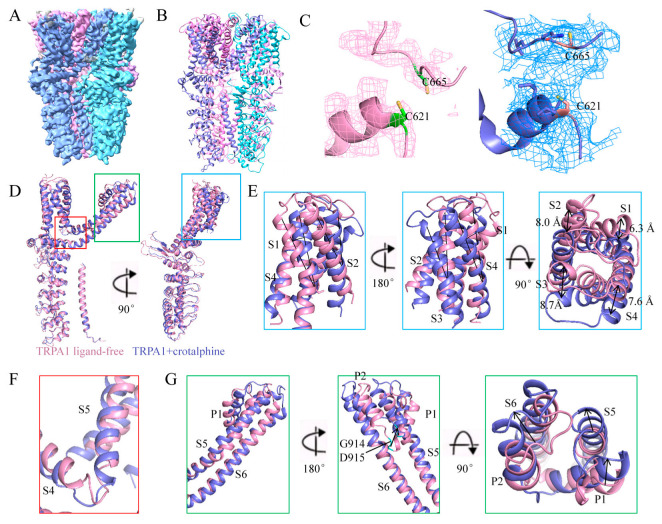
Structure of TRPA1 in complex with crotalphine. (**A**) The cryo-EM reconstruction map of TRPA1 in complex with crotalphine. The four subunits are shown in pink, blue, cyan and purple. (**B**) The structural model of TRPA1 in complex with crotalphine. The four subunits are shown in pink, blue, cyan and purple. (**C**) Local EM density maps of the Cys621 pocket. Left panel shows the ligand-free TRPA1 pocket, while the right panel displays the TRPA1-crotalphine complex pocket. (**D**) Structural superposition of the crotalphine-bound and ligand-free TRPA1 reveals conformational changes. (**E**) The structural superposition of the S1–S4 helices between crotalphine-bound and ligand-free TRPA1. (**F**) The structural superposition of the S4–S5 linker between crotalphine-bound and ligand-free TRPA1. (**G**) The structural superposition of the S5, S6 helices and pore domain between crotalphine-bound and ligand-free TRPA1.

Consistent with previous findings, we confirmed that crotalphine acts as an agonist of TRPA1 [30]. To resolve the structure of the crotalphine and TRPA1 complex, after constructing the complex in vitro, samples were flash-frozen and then we collected EM micrographs using a Titan Krios electron microscope. The 3D structure of the human TRPA1 with crotalphine binding was determined at a resolution of 3.8 Å (Figure 2A,B and Figure S3A,B). Notably, during atomic model building of the TRPA1-crotalphine complex, we observed significant unassigned electron density in the C621 binding pocket (Figure 2C), consistent with potential crotalphine binding at this site. However, the limited resolution of the electron density map in this region prevented unambiguous modeling and refinement of the crotalphine ligand.

Notable differences were observed between the overall structures of the complex and the ligand-free form, with a root mean square deviation (RMSD) of 2.974 Å for 552 Cα atoms. Structural alignment revealed that the coiled-coil domain and the cytoplasmic ankyrin repeat domain remain stationary, while the transmembrane region of the complex undergoes rotation and displacement relative to the stationary domains (Figure 2D). Specifically, the S1–S4 helices within the transmembrane domain of the complex structure exhibit a rotational displacement relative to their counterparts in the ligand-free structure (Figure 2E). Simultaneously, elongation and straightening of the S4–S5 linker and the S5 α-helix result in an upward shift and rotation of S5. Concomitantly, the S6 helix undergoes rotational movement coupled with upward displacement, resulting in concerted rotational and translational motions of the pore helices (P1 and P2) (Figure 2F,G).

**Figure 3:**
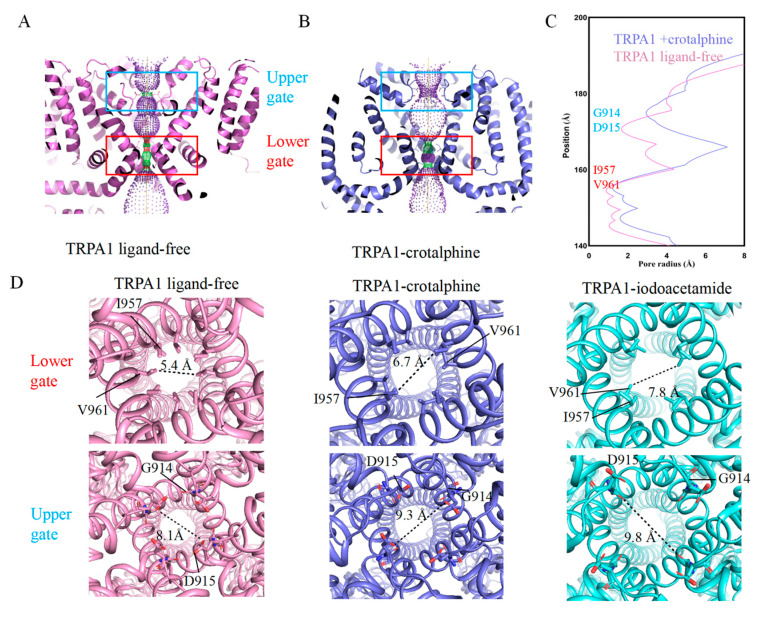

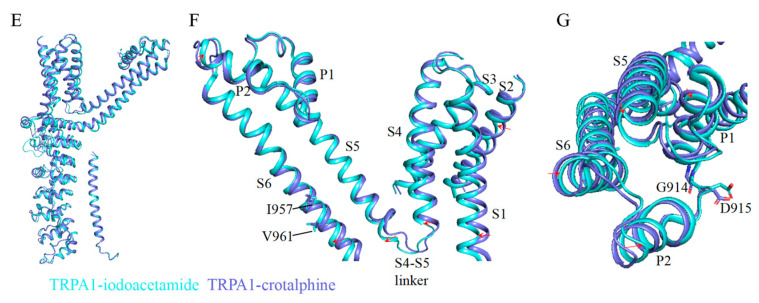
Changes in the pore region of the TRPA1 ion channel. (**A**) Cross-sectional view of TRPA1 in the ligand-free state, showing the upper and lower gates when the pore is in the closed state. (**B**) Cross-sectional view of TRPA1 in the crotalphine-bound state, showing the upper and lower gates when the pore is in the open state. (**C**) Schematic diagram of the pore radius of the TRPA1 ion channel. (**D**) Schematic representation of pore aperture dimensions at both upper and lower gates: ligand-free (close), crotalphine-bound, and iodoacetamide-bound (open) (PDB: 6V9X) states. (**E**) Structural superposition of TRPA1 in iodoacetamide-bound and crotalphine-bound states. (**F**) Comparison of transmembrane helices between iodoacetamide-bound and crotalphine-bound TRPA1. (**G**) Comparison of S5–S6 structures between iodoacetamide-bound and crotalphine-bound TRPA1.

The motion of transmembrane helices widens both the upper and lower gates of the ion permeation pathway. The upward movement and rotation of S6 expand the lower gate formed by residues I957 and V961. Simultaneously, the movement of the pore helices (P1 and P2) causes the upper gate, formed by residues G914 and D915 within the selectivity filter, to shift upward and dilate. A cross-sectional view of the TRPA1 ion permeation pathway reveals substantial changes in both the upper and lower gates (Figure 3A–C). Specifically, the pore diameter of the lower gate expands from 5.4 Å to 6.7 Å, while the upper gate widens from 8.1 Å to 9.3 Å. Comparative structural analysis reveals that the gate apertures in our TRPA1-crotalphine complex structure exhibit incomplete dilation, with neither the upper nor lower gates achieving the full expansion observed in the open-state conformation (iodoacetamide-bound TRPA1 structure, PDB: 6V9X) (Figure 3D). This structural observation is consistent with existing research, which suggests that crotalphine partially activates TRPA1 [30]. Therefore, we propose that the captured conformation likely represents an intermediate transitional state along the activation process.

By comparing the TRPA1 structures bound with iodoacetamide and crotalphine, we observed conformational differences in their transmembrane helices. In the crotalphine-bound structure, the S1–S6 helices do not undergo the complete rotational transition seen with iodoacetamide binding, and the S4–S5 linker fails to adopt the fully extended conformation observed in the iodoacetamide-bound state (Figure 3E,F). The lower gate, formed by I957 and V961 on the S6 helix, exhibits restricted dilation due to incomplete S6 helix rotation (Figure 3F). The upper gate is located on the loop between P1 and P2. In the crotalphine-bound structure, the S5 and S6 helices fail to rotate into a fully open state, preventing the loop between S5 and S6 from adopting its fully open conformation (Figure 3G). Consequently, the pore apertures of the upper and lower gates do not expand to the size observed in the fully open state.

### 3.3. Interaction Between Crotalphine and TRPA1

Previous studies have shown that electrophilic compounds activate the channel by covalently binding to the cysteine residues at the cytoplasmic amino-terminal of TRPA1. The cysteine residues (C621, C641 and C665 in human TRPA1) located at the membrane-proximal allosteric nexus are crucial for the binding of electrophiles and the activation of TRPA1 [20,22,23,35]. Bressan et al. identified three N-terminal cysteine residues (C621, C641, and C665) as potential primary binding sites for crotalphine through the application of whole-cell patch-clamp electrophysiology techniques [30]. In our complex map, a distinct excess density emerged in the C621 binding pocket. We suspect that this density corresponds to that of crotalphine. However, it is not clear enough to allow us to build the ligand into the map based on it.

**Figure 4:**
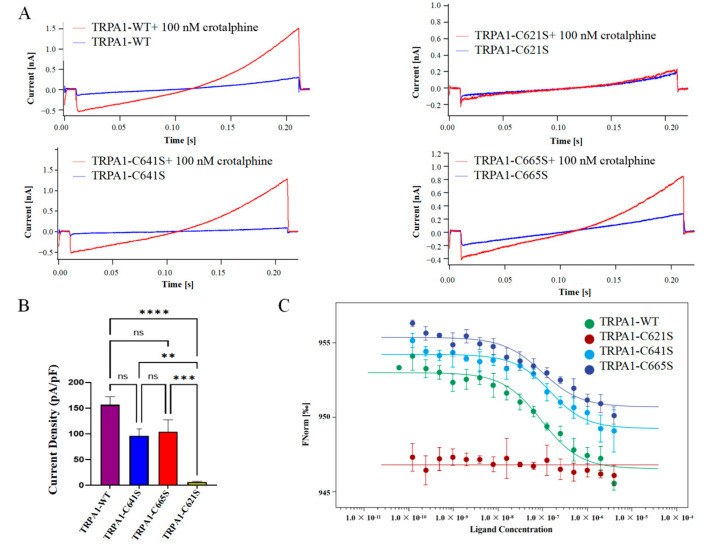
Sensitivity of TRPA1 channel residues C621, C641, and C665 to crotalphine. (**A**) Representative examples of current response of wild-type TRPA1 (WT) and its cysteine mutants (C621S, C665S, C641S) to 100 nM crotalphine application. (**B**) Current density (pA/pF) of wild-type and mutant TRPA1 channels (C621S, C641S, C665S) recorded at +120 mV in the presence of 100 nM crotalphine. Note significant reduction in crotalphine’s effect on TRPA1 displaying the C621S mutation (n = 9, +120 mV). *p*-values are denoted as follows: ** *p* < 0.01, *** *p* < 0.001, **** *p* < 0.0001, and ns not significant. (**C**) Analysis of the binding affinity between GFP-tagged TRPA1, TRPA1-C621S, TRPA1-C641S, TRPA1-C665S, and crotalphine using microscale thermophoresis (MST) technology. KdWT = 95.4 nM, KdC641S = 122.5 nM, KdC665S = 97.6 nM.

Therefore, to identify the specific binding sites of crotalphine on TRPA1, we generated single-point mutations at C621, C641, and C665 by substituting cysteine with serine, resulting in TRPA1 C621S, C641S, and C665S mutants, respectively. We first assessed the sensitivity of these mutants to crotalphine using patch-clamp recording. Electrophysiological data revealed that the C621S mutant exhibited no detectable currents upon crotalphine application, whereas the C641S and C665S mutants displayed current responses similar to those of the wild-type channel (Figure 4A,B). Next, we used MST to evaluate the binding affinity between crotalphine and the TRPA1 proteins (wild-type, C621S, C641S, and C665S). The MST results show that, compared with the wild-type TRPA1 protein, the interaction between C621S and crotalphine was significantly weakened, to the extent that the dissociation constant (Kd) could not be determined. In contrast, the Kd values for crotalphine binding to wild-type TRPA1, C641S, and C665S were 112.9 nM, 122.5 nM, and 116.6 nM, respectively (Figure 4C). These data indicate that mutating cysteine at positions 641 or 665 to serine does not significantly alter the interaction with crotalphine compared to the wild-type protein. Collectively, both mutation-based patch-clamp electrophysiology analysis and MST measurements support the conclusion that C621 is the primary binding site for crotalphine on TRPA1.

## 4. Discussion

In this study, we determined the cryo-EM structures of human TRPA1 in its ligand-free state and in complex with crotalphine at resolutions of 3.1 Å and 3.8 Å, respectively. We observed that TRPA1 in the ligand-free state adopts a closed conformation, whereas the crotalphine-bound complex adopts a desensitized state or an intermediate transitional state along the activation pathway. Notably, comparison of the two maps revealed an additional and distinct electron density in the C621 binding pocket of the crotalphine-bound complex. However, this density was insufficient to unambiguously fit the complete structure of crotalphine, suggesting that it may correspond to partial amino acid residues of crotalphine. To improve the resolution of individual subunits and binding pockets, we attempted to use the particle symmetry expansion, 3D classification, and local refinement in CryoSPARC software for further image processing [36]. Despite these efforts, the resolution of the crotalphine-bound complex did not improve significantly. We speculate that this limitation arises from the disruption of disulfide bonds at positions 7 and 14 of crotalphine upon binding to cysteine residues in TRPA1. This disruption likely renders crotalphine highly flexible, thereby preventing the reconstruction of a well-defined electron density map [31,37,38].

To identify the specific binding site of crotalphine on TRPA1, we first performed molecular docking simulations. However, these yielded inconsistent results, potentially due to the absence of a structural model for peptide-TRPA1 interactions; consequently, these data are not presented here. Therefore, we performed site-directed mutagenesis, substituting C621, C665, and C641 with serine. We then systematically evaluated the functional impact of these mutations on crotalphine binding using whole-cell patch-clamp recordings and MST analysis, which enabled the identification of critical binding residues. In general, both the mutation-based patch-clamp electrophysiology analysis and MST measurements confirmed that C621 is the primary binding site for crotalphine. In contrast, modifications at C641 and C665 were not essential for crotalphine-mediated channel activation, consistent with previous findings [22,23]. C621 is known to exhibit high reactivity in TRPA1 and serves as the primary site for electrophilic modification [21,22,23]. For instance, Suo et al. demonstrated that C621 is the primary site for covalent modification by electrophilic agonists such as BITC and JT010, while C665, located near the upper region of the C621 binding pocket, acts as a conformational switch to sense the covalent modification of C621 rather than being a direct target for modification [22]. Similarly, Zhao et al. reported that the small-molecule electrophilic reagent iodoacetamide requires modification of both C621 and C665 to stabilize the activated state of TRPA1. In contrast, its bulkier analog, BIA (BODIPY-iodoacetamide), can stabilize the activated state by modifying only C621, without the need for C665 modification [23]. In our study, crotalphine, a 14-amino acid peptide and a relatively large electrophilic reagent, similarly stabilizes the open state of TRPA1 through modification of C621 alone.

We attempt to propose the molecular mechanism by which crotalphine acts on TRPA1: crotalphine binding at the C621 allosteric site within the TRPA1 coupling domain, inducing rotational and translational movements of transmembrane helices that lead to cooperative dilation of both the extracellular vestibular gate and intracellular activation gate. These findings significantly advance our understanding of ligand-dependent TRPA1 gating mechanisms and provide crucial structural insights into the molecular basis of crotofoline-induced analgesic effects mediated by TRPA1 channel regulation.

## 5. Conclusions

Through combined patch-clamp electrophysiology and MST assays, we have identified C621 as the primary binding site for crotalphine on TRPA1. Single-particle cryo-EM analysis of the TRPA1-crotalphine complex revealed significant conformational rearrangements, particularly rotational movements of transmembrane helices, despite our inability to unambiguously model the crotalphine ligand in the density map. Structural measurements demonstrate that the pore aperture dimensions in this complex are intermediate between those of closed and open states. Based on these observations, we propose that the captured complex conformation likely represents a transitional intermediate along the gating pathway.

## Data Availability

Data described in the manuscript are contained within it. The EM data have been deposited in Electron Microscopy Data Bank (EMDB) with the accession code EMD-63720 and EMD-63715. The corresponding coordinates of TRPA1 have been deposited in the PDB with the accession code 9M8S and 9M8N.

## Supplementary Materials

The following supporting information can be downloaded at: https://www.mdpi.com/article/10.3390/membranes15060187/s1, Figure S1: Expression and purification of TRPA1 protein; Figure S2: Structure determination of the ligand-free TRPA1; Figure S3: Structure determination of TRPA1 in complex with crotalphine. Figure S4: The structures of the compounds.
