# Supplementary material for: Binding and Activating of Analgesic Crotalphine with Human TRPA1
Source: Membranes (Basel). 2025 Jun 19;15(6):187. doi: 10.3390/membranes15060187 (PMC12195256; doi:10.3390/membranes15060187)
Supplement: Supplementary file 1 [file membranes-15-00187-s001.zip › membranes-3659237-supplementary.pdf]

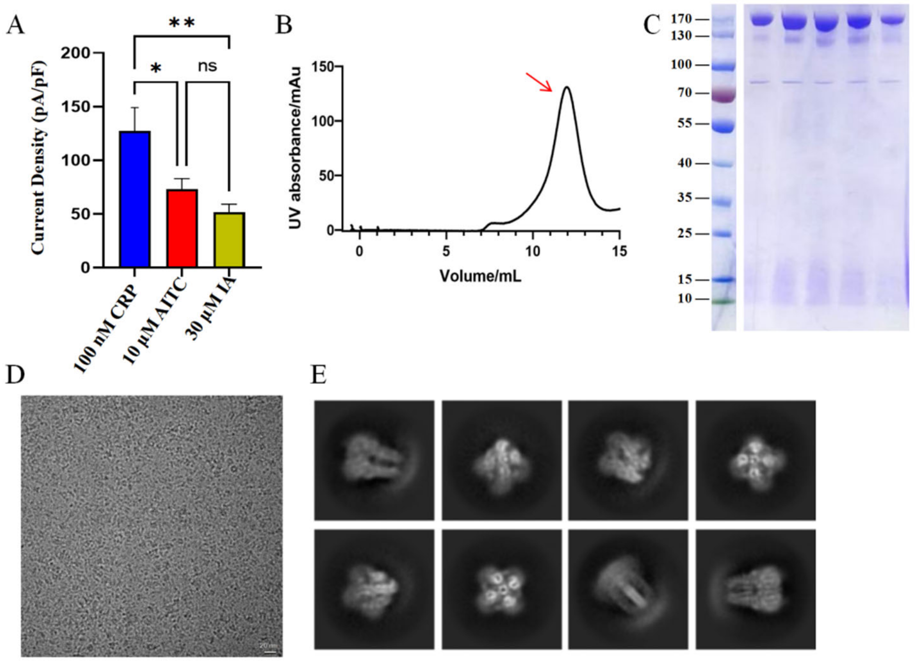

Supplementary Figure S1 Expression and purification of TRPA1 protein.

(A) Current densities (pA/pF) of wild-type TRPA1 in response to 100 nM crotalphine (CRP), 10 μM AITC, or 30 μM IA (n = 9, +120 mV). p-values are denoted as follows: *$p<0.05$, **$p<0.01$, and ns not significant.

(B) The size exclusion chromatography curve (Superose 6 increase 10/300 GL) of Full-length human TRPA1 protein purified by Strep-Tactin XT resin affinity chromatography.

(C) SDS-PAGE of the TRPA1 protein following its purification by size-exclusion chromatography.

(D) Representative cryo-EM micrographs of TRPA1.

(E) 2D classification averages of TRPA1.

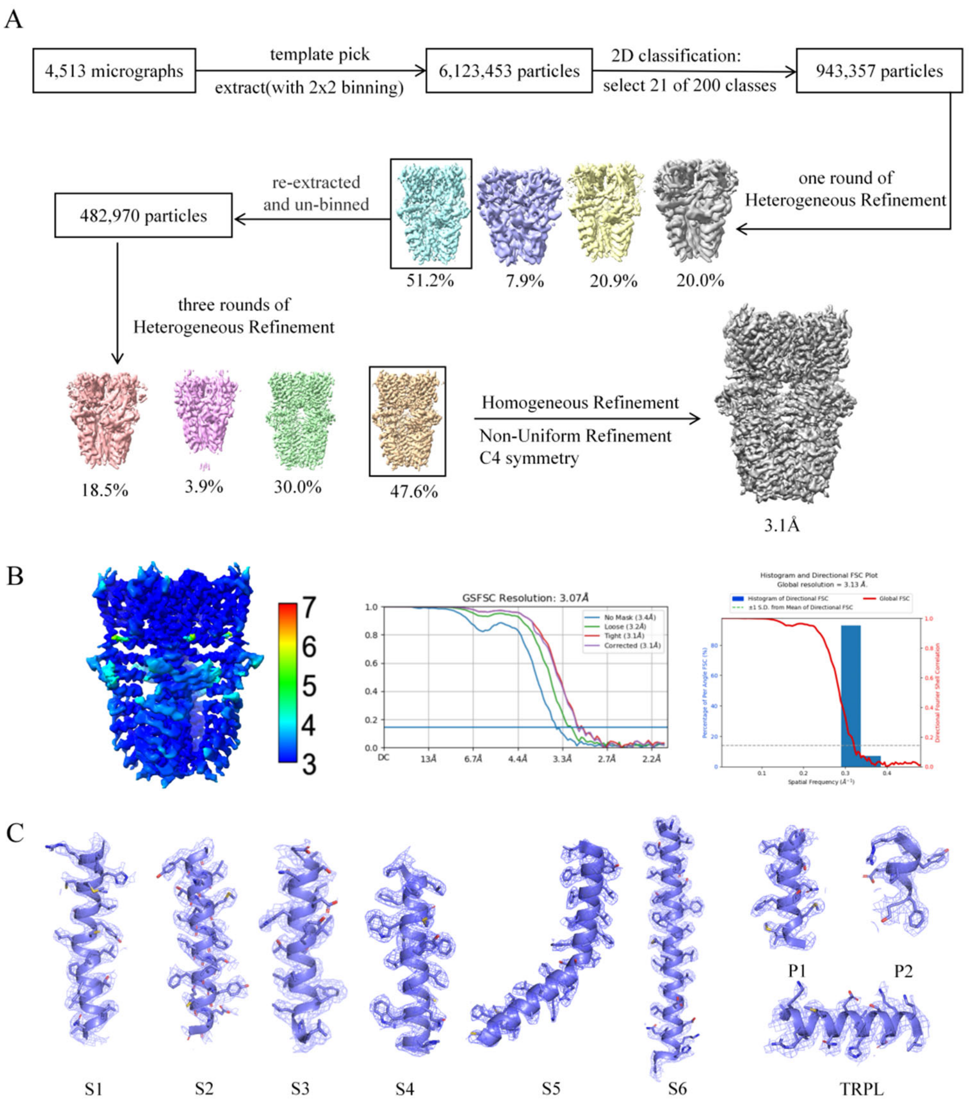

Supplementary Figure S2 Structure determination of the ligand-free TRPA1.

(A) The flow chart of cryo-EM data processing of the ligand-free TRPA1. The overall resolution of the ligand-free TRPA1 was determined at 3.1 Å.

(B) Local resolution, GSFSC curve and 3D FSC curve of ligand-free TRPA1.

(C) Local EM density of transmembrane helices and P1, P2, TRPL helices of the ligand-free TRPA1.

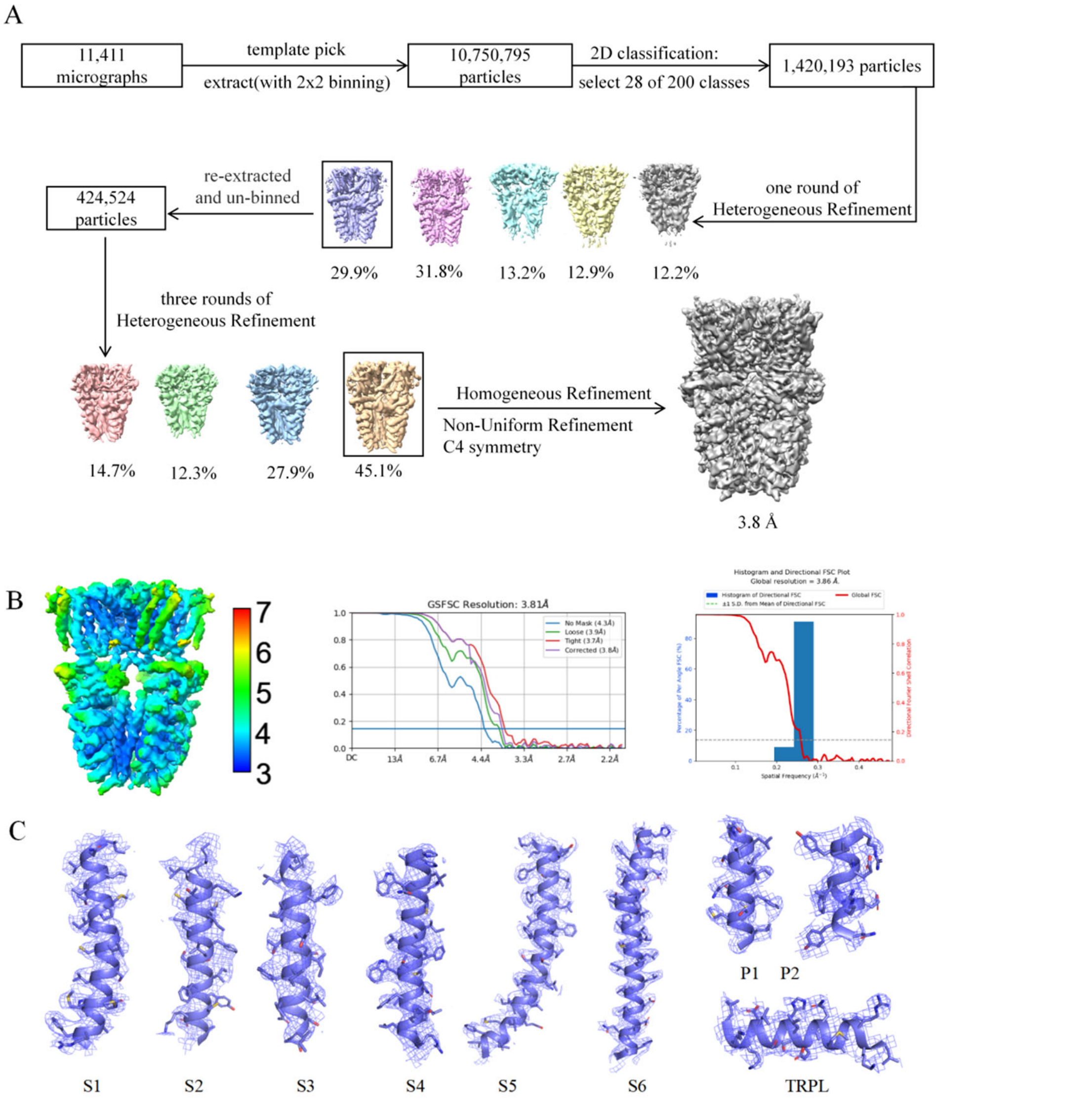

Supplementary Figure S3 Structure determination of TRPA1 in complex with crotalphine.

(A) The flow chart of cryo-EM data processing of TRPA1 in complex with crotalphine. The overall resolution of the TRPA1 in complex with crotalphine was determined at 3.1 Å.

(B) Local resolution, GSFSC curve and 3D FSC curve of TRPA1 in complex with crotalphine.

(C) EM density of transmembrane helices and P1, P2, TRPL helices of the TRPA1 in complex with crotalphine.

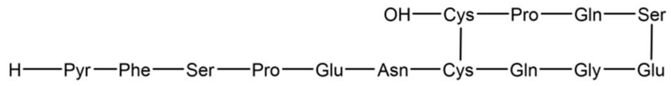

crotalphine

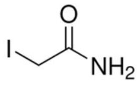

iodoacetamide (IA)

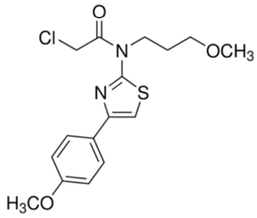

JT010

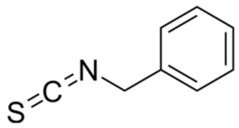

Benzyl isothiocyanate (BITC)

Supplementary Figure S4 The structures of the compounds.
